# Universal Tool for Single-Photon Circuits: Quantum Router Design

**DOI:** 10.3390/ma13020319

**Published:** 2020-01-10

**Authors:** Aydar Sultanov, Yakov Greenberg, Evgeniya Mutsenik, Dmitry Pitsun, Evgeni Il’ichev

**Affiliations:** 1 Faculty of Physical Engineering, Novosibirsk State Technical University, Novosibirsk 630073, Russia; yakovgreenberg@yahoo.com (Y.G.); mutsenik.e@gmail.com (E.M.); dmitrijjpicun@rambler.ru (D.P.); 2 Leibniz Institute of Photonic Technology, 07745 Jena, Germany; evgeni.ilichev@leibniz-ipht.de; 3 Russian Quantum Center, Skolkovo 121205, Russia

**Keywords:** quantum routing, microwave circuits, open waveguide, transmission line, quantum measurements

## Abstract

We demonstrate that the non-Hermitian Hamiltonian approach can be used as a universal tool to design and describe a performance of single photon quantum electrodynamical circuits (cQED). As an example of the validity of this method, we calculate a novel six port quantum router, constructed from four qubits and three open waveguides. We have obtained analytical expressions, which describe the transmission and reflection coefficients of a single photon in general form taking into account the spread qubit’s parameters. We show that, due to naturally derived interferences, in situ tuning the probability of photon detection in desired ports.

## 1. Introduction

Quantum circuits represent an important part of a rapidly developing research area, which includes the quantum information transfer and processing. Photons propagation in these circuits is associated with the very low coherence losses even for long distances, which makes them the prime contender for the quantum information carriers. The manipulation of a single photon is the key issue for the performance of quantum systems [1]. A modern technology allows for realizing many-qubit quantum circuits both in optical and microwave frequency ranges. Due to a relatively big scale of microwave quantum elements, especially qubits, a coupling between artificial quantum systems and photons can be easily implemented. Indeed, even strong and ultra-strong regimes have been already demonstrated [2,3,4,5].

In order to describe the performance of these circuits, several theoretical approaches, developed in quantum optics and nuclear physics, are applied. The mostly used ones are the master equation approach, input–output theory, a solution of Schrödinger equation in the configuration space. A set of microwave devices: quantum gates, sources and detectors of single photons, quantum routers, arbitrary number generators, and so on have been successfully analyzed by these approaches. Basically, these methods were derived for natural atoms with indistinguishable parameters. However, solid-state based qubits have unavoidable technological parameters spread. This spread leads to the discrepancies of the qubits’ eigenfrequencies and their coupling coefficients to the microwave fields. Additionally, the boundary conditions should be specified taking into account the distances between qubits [6]. Therefore, there is no straightforward mapping between quantum optics and microwave circuits’ quantum electrodynamics. For example, the input–output theory, successfully implemented for the problems with one or two scatters [7,8,9,10], fails in the describing of complex circuits with multiple scatterers. The reason here is the appearing of the non-Markovian photon dynamics which requires the formalism modification [11].

Recently, the non-Hermitian Hamiltonian approach, successfully implemented in nuclear physics [12,13], have been adapted for the problems of a single photon scattering in microwave quantum circuits [14]. In general, this approach allows for obtaining the scattering and steady-state parameters of any microwave cQED chain. It was successfully applied to describe a single photon transport through the one and two qubit structures [14,15,16,17,18,19]. In the frame of this approach, the parameters spread and distances are easily being taken into account. Here, circuits are represented as a set of states with discrete and continuous energy spectra, interconnected through the decay channels (see details in [14]). In this paper, we demonstrate the applicability of this method for cQED by calculation of a six-port quantum router. Note that the calculated circuit has the highest number of ports among devices that have been considered so far.

The paper is arranged as follows. In Section 1, we present the description of a quantum router. In Section 2, we show a theoretical description of a router in terms of a non-Hermitian Hamiltonian approach. In Section 3, we focus on the analytical equations for routing probabilities and steady state wave function. Here, we reconstruct the scattering coefficients and analyze some limit cases for the scattering parameters. In Section 4, we demonstrate the control probabilities through qubits’ eigenfrequencies tuning. Finally, in conclusion, we summarize our results and discuss the possible research directions that could be done on the basis of our results.

## 2. Quantum Router

The quantum router is used to control a single-photon propagation into desired ports. It means that a designing of a quantum router for the practical application is the problem of a single photon scattering. The main requirements for quantum routers of single photons are formulated in [20]: (1) both the signal and control information have to be stored in quantum objects; (2) the signal has to be unchanged under the routing operation; (3) the router has to be able to route the signal into a coherent superposition; (4) the router has to work with no need for post-selection; and, (5) in order to optimize the resources of the quantum network, every individual qubit has to control a single photon signal. For the usable device, we want to add the requirement of a broadband access, which is impossible to realize for the system where resonators and cavities are being used [7,9,10,21,22,23,24,25,26]. Several types of the router were successfully realized in the optical frequency range [7,8,9,10,21,22,23,24,27,28,29], and some models, based on quantum optics approaches, were proposed for the microwave frequency range [25,26,30,31,32]. Basically, the routers could be divided into two main groups: exploiting multilevel atoms [8,22,24,26] and two-level quantum systems [9,10,25,28,30,31,32]. Similar devices were also investigated for a 3D optic lattice [29], implementing nanomechanical systems [21], as a combination of standard quantum gates [20], and using the chirality (coupling between atom and field is direction-dependent) [33]. For more details, one could follow a review paper [34].

Theoretically, most of the structures were described by making use of the standard methods: direct solving of Schrödinger equations or Heisenberg representation approach. In the first case, the problem of the systems with three (or less) elements (qubits+cavities+waveguides) is solved by the discretization of waveguides (constructed from coupled resonance cavities) [22,26,31]. For this case, the Laplace transformations [6] or the real-space approach [24,28] are used. In the Heisenberg representation approach, the input–output theory is utilized for similar systems with a modest complexity [7,8,9,10,23,25]. It worth mentioning that results of numerically solved master-equations for the device constructed of two scatterers, waveguide, and two-mode cavity (considered as five element system), are presented in [7].

**Figure 1:**
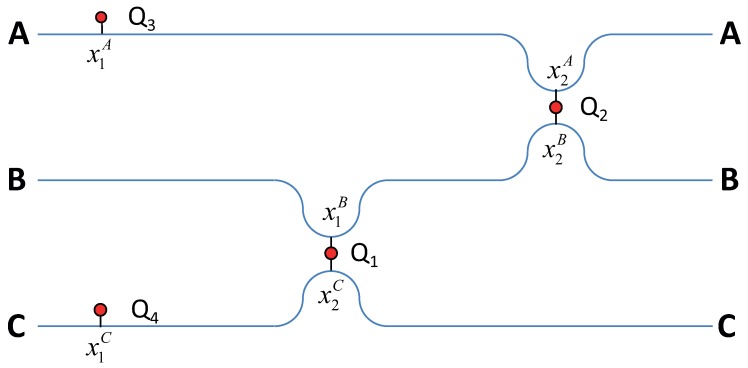
The scheme of quantum router: sketch of the system constructed from three waveguides and four qubits at coordinates $x_{I}^{J}$, where *I* means position at the $J^{th}$ waveguide (J=A,B,C).

It is well known that two waveguides could be coupled with each other through the qubit [25]. The propagating photon is resonantly absorbed by the qubit, and the radiation probability back to the waveguide depends on the coupling between the waveguides and the qubit. In addition, it is well known that, in an open waveguide, the qubit behaves as a reflector for a photon with a frequency equal to the qubit’s eigenfrequency [35]. This property could be used to control the photon transmission in the waveguide, when the qubit plays a role of a quantum switch. If we place two qubits into the single waveguide, the photon transmission and reflection will depend on the interference between the wavefunctions, corresponding to interactions with the first and second qubits [16]. This interference depends on both the distance between qubits and their eigenfrequencies. Exploiting these features, we offer the router device, which presents three waveguides and four qubits (see Figure 1). Two qubits Q1 (eigenfrequency $\Omega_{1}$) and Q2 (eigenfrequency $\Omega_{2}$) play the role of interconnectors between the waveguide *B* and the waveguides *A*, *C*. Qubits Q3 (eigenfrequency $\Omega_{3}$) and Q4 (eigenfrequency $\Omega_{4}$) play the role of auxiliary reflectors to realize the interference conditions. In the solid-state qubits, the individual control of eigenfrequencies is realizable. It means that one could tune the interaction between the qubit and photons, and thus control the interference by proper adjustment of the distances.

A similar scheme was proposed in the theoretical work [25], where the interaction between open waveguides is controlled by a qubit. However, the main advantages of our device are the accounting for the distances between qubits, clear scalability, near unity routing quality, the opportunity to create a coherent superposition state, and the absence of necessity to use additional circulators, which are required in all works with the open waveguides. Thus, the last advantage comes from using the auxiliary scatterers and automatically fulfills condition 4 from [20]. Additionally, an absence of any cavities and circulators provides a required broadband range.

## 3. Results

### 3.1. The System and Method Description

The full Hamiltonian of the system is written as follows:

(1) $$\begin{array}{c} \hat{H}=\sum_{k,l=a,b,c}\hbar \omega_{k}^{l}{\hat{l}}_{k}^{†}{\hat{l}}_{k}^{}+\sum_{i=1}^{4}\frac{1}{2}\hbar \Omega_{i}\left(1+{\hat{\sigma }}_{z}^{\left(i\right)}\right)+\sum_{k}^{}\hbar \xi_{A}^{3}\left({\hat{a}}_{k}^{†}{\hat{\sigma }}_{-}^{\left(3\right)}e^{-jkx_{1}^{A}}+{\hat{a}}_{k}{\hat{\sigma }}_{+}^{\left(3\right)}e^{jkx_{1}^{A}}\right)\\ +\sum_{k}^{}\hbar \xi_{A}^{2}\left({\hat{a}}_{k}^{†}{\hat{\sigma }}_{-}^{\left(2\right)}e^{-jkx_{2}^{A}}+{\hat{a}}_{k}{\hat{\sigma }}_{+}^{\left(2\right)}e^{jkx_{2}^{A}}\right)+\sum_{k}^{}\hbar \xi_{B}^{1}\left({\hat{b}}_{k}^{†}{\hat{\sigma }}_{-}^{\left(1\right)}e^{-jkx_{1}^{B}}+{\hat{b}}_{k}{\hat{\sigma }}_{+}^{\left(1\right)}e^{jkx_{1}^{B}}\right)\\ +\sum_{k}^{}\hbar \xi_{B}^{2}\left({\hat{b}}_{k}^{†}{\hat{\sigma }}_{-}^{\left(2\right)}e^{-jkx_{2}^{B}}+{\hat{b}}_{k}{\hat{\sigma }}_{+}^{\left(2\right)}e^{jkx_{2}^{B}}\right)+\sum_{k}^{}\hbar \xi_{C}^{1}\left({\hat{c}}_{k}^{†}{\hat{\sigma }}_{-}^{\left(4\right)}e^{-jkx_{1}^{C}}+{\hat{c}}_{k}{\hat{\sigma }}_{+}^{\left(4\right)}e^{jkx_{1}^{C}}\right)+\\ \sum_{k}^{}\hbar \xi_{C}^{2}\left({\hat{c}}_{k}^{†}{\hat{\sigma }}_{-}^{\left(1\right)}e^{-jkx_{2}^{C}}+{\hat{c}}_{k}{\hat{\sigma }}_{+}^{\left(1\right)}e^{jkx_{2}^{C}}\right), \end{array}$$

where ${\hat{l}}_{k}^{†}\left({\hat{l}}_{k}^{}\right)$ are the bosonic operators of creation (annihilation) photons with wave vector *k* (and with a frequency $\omega_{k}^{l}$) in $l^{th}$ waveguide, i.e., $\hat{a}$, $\hat{b}$, $\hat{c}$ corresponds to the A, B and C waveguides; ${\hat{\sigma }}_{z}^{\left(i\right)}\;=\;|e_{i}\rangle \langle e_{i}|-|g_{i}\rangle \langle g_{i}|$ - Pauli spin operators, where $|e_{i}\rangle$ ($|g_{i}\rangle$) are the excited (ground) state of $i^{th}$ qubit; the interaction between waveguide photons and qubits are described through the Jaynes-Cummings model, where ${\hat{\sigma }}_{-}^{\left(i\right)}=|g_{i}\rangle \langle e_{i}|$ (${\hat{\sigma }}_{+}^{\left(i\right)}=|e_{i}\rangle \langle g_{i}|$ ) are lowering (raising) operators for $i^{th}$ qubit and $\xi_{J}^{i}$ is the coupling between $J^{th}$ waveguide and $i^{th}$ qubit; *ℏ* is the Planck constant, and hereafter we take ħ=1. Because in the open waveguides the energy spectrum is continuous, the summation over *k* should be replaced by the integration as

$$\sum_{k}\to \frac{L}{2\pi }\int_{-\infty }^{+\infty }dk,$$

where *L* is a waveguide’s length, and for the simplicity we will formally write the summation sign. For one photon routing, we restrict the states basis to the one-excitation states, so we introduce them as follows:

(2) $$\begin{array}{c} |A\rangle =|k_{A}{,0}_{B}{,0}_{C}\rangle ⊗|G\rangle ,\\ |B\rangle =|0_{A},k_{B}{,0}_{C}\rangle ⊗|G\rangle ,\\ |C\rangle =|0_{A}{,0}_{B},k_{C}\rangle ⊗|G\rangle , \end{array}$$

where the $|G\rangle =|g_{1},g_{2},g_{3},g_{4}\rangle$ is the ground state of all qubits, so $|J\rangle$ (J=A,B,C) corresponds to the situation when there is one photon in $J^{th}$ waveguide and all qubits in the ground states. Then, this single photon can be absorbed by one of four qubits, leaving the waveguides empty, so we define these states as:

(3) $$\begin{array}{c} |1\rangle =|e_{1},g_{2},g_{3},g_{4}\rangle ⊗|0_{vac}\rangle ,\\ |2\rangle =|g_{1},e_{2},g_{3},g_{4}\rangle ⊗|0_{vac}\rangle ,\\ |3\rangle =|g_{1},g_{2},e_{3},g_{4}\rangle ⊗|0_{vac}\rangle ,\\ |4\rangle =|g_{1},g_{2},g_{3},e_{4}\rangle ⊗|0_{vac}\rangle , \end{array}$$

where $|0_{vac}\rangle$ is the photonic vacuum state (such that $l_{k}^{†}|0_{vac}\rangle =|k_{J}\rangle$ respectively for each waveguide), and $|i\rangle$ (where i=1...4) describes the state when the $i^{th}$ qubit is excited.

It is obvious that states (2) have a continuous energy spectrum, due to arbitrary photon’s frequency in an open waveguide, and states (3) have a discrete energy spectrum. It allows us to define two different groups of Hilbert space’ states with a continuous and discrete spectrum. This subdivision is proper for an easy calculation in the frame of the non-Hermitian Hamiltonian approach (but generally, any subdivision of the space is possible). This way, following the approach [14], we introduce the projection operators:

(4) $$\begin{array}{c} \hat{P}=\frac{L_{A}}{2\pi }\int dk_{A}|A\rangle \langle A|+\frac{L_{B}}{2\pi }\int dk_{B}|B\rangle \langle B|\\ +\frac{L_{C}}{2\pi }\int dk_{C}|C\rangle \langle C|,\\ \hat{Q}=|1\rangle \langle 1|+|2\rangle \langle 2|+|3\rangle \langle 3|+|4\rangle \langle 4|, \end{array}$$

which obey the following equations $\hat{P}\hat{Q}=\hat{Q}\hat{P}=0;\hat{P}\hat{P}=\hat{P};\hat{Q}\hat{Q}=\hat{Q};\hat{P}+\hat{Q}=1$ and $L_{J}$ (J=A,B,C) are the corresponding waveguides’ lengths.

We want to describe probabilities of transitions between states (2) through all trajectories including internal states (3). The effective non-Hermitian Hamiltonian is defined on the basis of internal states (3), describing the decay of these states due to coupling to the continuum. Here, we leave out routine calculations, which are similar to ones from [14], and write the effective Hamiltonian and full system’s wave functions as follows:

(5) $${\hat{H}}_{eff}={\hat{H}}_{QQ}+{\hat{H}}_{QP}\frac{1}{E-{\hat{H}}_{PP}+i\varepsilon }{\hat{H}}_{PQ},$$

(6) $$|\Psi \rangle =|in\rangle +\frac{1}{E-{\hat{H}}_{eff}}{\hat{H}}_{QP}|in\rangle +\frac{1}{E-{\hat{H}}_{PP}}{\hat{H}}_{PQ}\frac{1}{E-{\hat{H}}_{eff}}{\hat{H}}_{QP}|in\rangle ,$$

where ${\hat{H}}_{XY}=\hat{X}\hat{H}\hat{Y}$ ($\hat{X},\hat{Y}=\hat{P},\hat{Q}$) is the projection of the full Hamiltonian (1) and $|in\rangle$ presents the system’s state before the photon scattered at multiqubit internal system, which can be expressed through the states (3) with defined initial wave vector $k_{0}=\frac{\omega }{\nu_{g}}$ (hereafter $\omega =\omega_{k}^{l}$ is frequency and $\nu_{g}$ is the group velocity of the scattering photon) and initial state’s energy *E*, i.e., $|in\rangle =|A\rangle ,|B\rangle ,|C\rangle$. Since the chosen basis of states is supposed to be full, we use the fullness property $\hat{P}+\hat{Q}=1$:

$$|\Psi \rangle =|in\rangle +\frac{1}{E-{\hat{H}}_{eff}}\left(\hat{P}+\hat{Q}\right){\hat{H}}_{QP}|in\rangle +\frac{1}{E-{\hat{H}}_{PP}}\left(\hat{P}+\hat{Q}\right){\hat{H}}_{PQ}\left(\hat{P}+\hat{Q}\right)\frac{1}{E-{\hat{H}}_{eff}}\left(\hat{P}+\hat{Q}\right){\hat{H}}_{QP}|in\rangle .$$

Here, the second term describes only the internal system behavior, and the third term describes transitions between external states, defined by the trajectories through the internal states. The internal states behavior is out of the paper’s scope because only the external states with the photon in the waveguides are detectable. Considering Equation (4), one gets the following form of the full system’s wave function:

(7) $$|\Psi \rangle =|in\rangle +\sum_{n,m=1}^{4}|n\rangle R_{nm}\langle m|{\hat{H}}_{QP}|in\rangle +\sum_{J=A,B,C}\sum_{n,m=1}^{4}\int dk_{J}\frac{|k_{J}\rangle }{E-E_{J}\left(k_{J}\right)}\langle J|{\hat{H}}_{PQ}|n\rangle \cdot \cdot R_{nm}\langle m|{\hat{H}}_{QP}|in\rangle ,$$

where $R_{mn}=\langle m|\frac{1}{E-H_{eff}}|n\rangle$. Summation over *J* in Equation (7) shows that, for each initial state $|in\rangle$, there are some probabilities to be transformed to one of three different final states. Summation over n,m describes the probabilities of these transformations, defined by the interaction with internal states.The strength of these interactions depends on couplings between the internal and initial external states (term $\langle m|{\hat{H}}_{QP}|in\rangle$), between internal and external states in general (terms $\langle J|{\hat{H}}_{PQ}|n\rangle$) and also depends on the effective interaction between the internal states (term $R_{nm}$). The effective Hamiltonian of the system could be expressed in the matrix form in the basis of states (3) as:

(8) $${\hat{H}}_{eff}=\left(\begin{array}{cccc} \Omega_{1}-j\Gamma_{B1}-j\Gamma_{C1} & -j\sqrt{\Gamma_{B1}\Gamma_{B2}}e^{jk\left|x_{1}^{B}-x_{2}^{B}\right|} & 0 & -j\sqrt{\Gamma_{C1}\Gamma_{C4}}e^{jk\left|x_{1}^{C}-x_{2}^{C}\right|}\\ -j\sqrt{\Gamma_{B1}\Gamma_{B2}}e^{jk\left|x_{1}^{B}-x_{2}^{B}\right|} & \Omega_{2}-j\Gamma_{B2}-j\Gamma_{A2} & -j\sqrt{\Gamma_{A1}\Gamma_{A3}}e^{jk\left|x_{1}^{A}-x_{2}^{A}\right|} & 0\\ 0 & -j\sqrt{\Gamma_{A1}\Gamma_{A3}}e^{jk\left|x_{1}^{A}-x_{2}^{A}\right|} & \Omega_{3}-j\Gamma_{A3} & 0\\ -j\sqrt{\Gamma_{C1}\Gamma_{C4}}e^{jk\left|x_{1}^{C}-x_{2}^{C}\right|} & 0 & 0 & \Omega_{4}-j\Gamma_{C4} \end{array}\right),$$

where $\Gamma_{Ji}$( i=1…4) describes the $i^{th}$ qubit decay rate to the $J^{th}$ waveguide, and it can be expressed through the couplings $\Gamma_{Ji}=\frac{L_{J}{\left(\xi_{J}^{i}\right)}^{2}}{v_{g}^{J}}$, where $L_{J}$ and $v_{g}^{J}$ are the length of $J^{th}$ waveguide and photon wave’s group velocity in $J^{th}$ waveguide with a linear dispersion, consequently. In the next section, the wavefunctions of the system are given.

### 3.2. Solutions and Transmission Probabilities

Generally, the quantum router distributes the detection probability between different ports. The detection probability is described by the wavefunction. The non-Hermitian Hamiltonian approach allows for easily finding these wavefunctions. Because the initial state could be prepared as one of three states (see Equation (2)), according to Equation (7), we get three different final wave functions per each initial state—for example, the observation probability of the photon in the waveguide *B*, which was initially sent to *A*, defined by the function $\langle x_{B}|\Psi_{A}\rangle$. These wave functions, after some routine algebra, omitting details, allow us to find nine solutions in the configuration space:

(9) $$\begin{array}{c} \langle x_{A}|\Psi_{A}\rangle =e^{jk_{0}x}-j\sqrt{\Gamma_{A2}}e^{jk_{0}x_{2}^{A}}\left(R_{22}\sqrt{\Gamma_{A2}}e^{jk_{0}\left|x-x_{2}^{A}\right|}+R_{32}\sqrt{\Gamma_{A3}}e^{jk_{0}\left|x-x_{1}^{A}\right|}\right)\\ -j\sqrt{\Gamma_{A3}}e^{jk_{0}x_{1}^{A}}\left(R_{23}\sqrt{\Gamma_{A2}}e^{jk_{0}\left|x-x_{2}^{A}\right|}+R_{33}\sqrt{\Gamma_{A3}}e^{jk_{0}\left|x-x_{1}^{A}\right|}\right); \end{array}$$

(10) $$\begin{array}{c} \langle x_{B}|\Psi_{A}\rangle =-j\sqrt{\Gamma_{A2}}e^{jk_{0}x_{2}^{A}}\left(R_{12}\sqrt{\Gamma_{B1}}e^{jk_{0}\left|x-x_{1}^{B}\right|}+R_{22}\sqrt{\Gamma_{B2}}e^{jk_{0}\left|x-x_{2}^{B}\right|}\right)\\ -j\sqrt{\Gamma_{A3}}e^{jk_{0}x_{1}^{A}}\left(R_{13}\sqrt{\Gamma_{B1}}e^{jk_{0}\left|x-x_{1}^{B}\right|}+R_{23}\sqrt{\Gamma_{B2}}e^{jk_{0}\left|x-x_{2}^{B}\right|}\right); \end{array}$$

(11) $$\begin{array}{c} \langle x_{C}|\Psi_{A}\rangle =-j\sqrt{\Gamma_{A2}}e^{jk_{0}x_{2}^{A}}\left(R_{12}\sqrt{\Gamma_{C1}}e^{jk_{0}\left|x-x_{2}^{C}\right|}+R_{42}\sqrt{\Gamma_{C4}}e^{jk_{0}\left|x-x_{1}^{C}\right|}\right)\\ -j\sqrt{\Gamma_{A3}}e^{jk_{0}x_{1}^{A}}\left(R_{13}\sqrt{\Gamma_{C1}}e^{jk_{0}\left|x-x_{2}^{C}\right|}+R_{43}\sqrt{\Gamma_{C4}}e^{jk_{0}\left|x-x_{1}^{C}\right|}\right); \end{array}$$

(12) $$\begin{array}{c} \langle x_{A}|\Psi_{B}\rangle =-j\sqrt{\Gamma_{B1}}e^{jk_{0}x_{1}^{B}}\left(R_{21}\sqrt{\Gamma_{A2}}e^{jk_{0}\left|x-x_{2}^{A}\right|}+R_{31}\sqrt{\Gamma_{A3}}e^{jk_{0}\left|x-x_{1}^{A}\right|}\right)\\ -j\sqrt{\Gamma_{B2}}e^{jk_{0}x_{2}^{B}}\left(R_{22}\sqrt{\Gamma_{A2}}e^{jk_{0}\left|x-x_{2}^{A}\right|}+R_{32}\sqrt{\Gamma_{A3}}e^{jk_{0}\left|x-x_{1}^{A}\right|}\right); \end{array}$$

(13) $$\begin{array}{c} \langle x_{B}|\Psi_{B}\rangle =e^{jk_{0}x}-j\sqrt{\Gamma_{B1}}e^{jk_{0}x_{1}^{B}}\left(R_{11}\sqrt{\Gamma_{B2}}e^{jk_{0}\left|x-x_{1}^{B}\right|}+R_{21}\sqrt{\Gamma_{B1}}e^{jk_{0}\left|x-x_{2}^{B}\right|}\right)\\ -j\sqrt{\Gamma_{B2}}e^{jk_{0}x_{2}^{B}}\left(R_{12}\sqrt{\Gamma_{B1}}e^{jk_{0}\left|x-x_{1}^{B}\right|}+R_{22}\sqrt{\Gamma_{B2}}e^{jk_{0}\left|x-x_{2}^{B}\right|}\right); \end{array}$$

(14) $$\begin{array}{c} \langle x_{C}|\Psi_{B}\rangle =-j\sqrt{\Gamma_{B1}}e^{jk_{0}x_{1}^{B}}\left(R_{11}\sqrt{\Gamma_{C1}}e^{jk_{0}\left|x-x_{2}^{C}\right|}+R_{41}\sqrt{\Gamma_{C4}}e^{jk_{0}\left|x-x_{1}^{C}\right|}\right)\\ -j\sqrt{\Gamma_{B2}}e^{jk_{0}x_{2}^{B}}\left(R_{12}\sqrt{\Gamma_{C1}}e^{jk_{0}\left|x-x_{2}^{C}\right|}+R_{42}\sqrt{\Gamma_{C4}}e^{jk_{0}\left|x-x_{1}^{C}\right|}\right); \end{array}$$

(15) $$\begin{array}{c} \langle x_{A}|\Psi_{C}\rangle =-j\sqrt{\Gamma_{C1}}e^{jk_{0}x_{2}^{C}}\left(R_{21}\sqrt{\Gamma_{A2}}e^{jk_{0}\left|x-x_{2}^{A}\right|}+R_{31}\sqrt{\Gamma_{A3}}e^{jk_{0}\left|x-x_{1}^{A}\right|}\right)\\ -j\sqrt{\Gamma_{C4}}e^{jk_{0}x_{1}^{C}}\left(R_{24}\sqrt{\Gamma_{A2}}e^{jk_{0}\left|x-x_{2}^{A}\right|}+R_{34}\sqrt{\Gamma_{A3}}e^{jk_{0}\left|x-x_{1}^{A}\right|}\right); \end{array}$$

(16) $$\begin{array}{c} \langle x_{B}|\Psi_{C}\rangle =-j\sqrt{\Gamma_{C1}}e^{jk_{0}x_{2}^{C}}\left(R_{11}\sqrt{\Gamma_{B2}}e^{jk_{0}\left|x-x_{1}^{B}\right|}+R_{21}\sqrt{\Gamma_{B1}}e^{jk_{0}\left|x-x_{2}^{B}\right|}\right)\\ -j\sqrt{\Gamma_{B2}}e^{jk_{0}x_{2}^{B}}\left(R_{14}\sqrt{\Gamma_{B1}}e^{jk_{0}\left|x-x_{1}^{B}\right|}+R_{24}\sqrt{\Gamma_{B2}}e^{jk_{0}\left|x-x_{2}^{B}\right|}\right); \end{array}$$

(17) $$\begin{array}{c} \langle x_{C}|\Psi_{C}\rangle =e^{jk_{0}x}-j\sqrt{\Gamma_{C1}}e^{jk_{0}x_{2}^{C}}\left(R_{11}\sqrt{\Gamma_{C1}}e^{jk_{0}\left|x-x_{2}^{C}\right|}+R_{41}\sqrt{\Gamma_{C4}}e^{jk_{0}\left|x-x_{1}^{C}\right|}\right)\\ -j\sqrt{\Gamma_{C4}}e^{jk_{0}x_{1}^{C}}\left(R_{14}\sqrt{\Gamma_{C1}}e^{jk_{0}\left|x-x_{2}^{C}\right|}+R_{44}\sqrt{\Gamma_{C4}}e^{jk_{0}\left|x-x_{1}^{C}\right|}\right), \end{array}$$

where we have introduced waveguide photon’s state in the configuration basis and used $\langle x_{n}|k_{m}\rangle \;=\;\delta_{mn}e^{jk_{m}x_{n}}$, which is raised from definitions of photon states, for example $|k_{A}\rangle \;=\;a_{k}^{†}|0\rangle$ and $|x_{A}\rangle =\sum_{k}a_{k}^{†}e^{ikx_{A}}|0\rangle ⊗|G\rangle$. Henceforward, we will omit these indices of *k* and *x* to not overload the equations, and we will comment on it when it is needed.

Equation (9) describes the wave function, which can be used to detect the photon in *A* waveguide (independently of its direction) if the photon was initially sent to this waveguide *A*. The first term just refers to a wave of initially sent photons. If we suppose that there is no any interaction between this photon and qubits Q2 and Q3 ($\Gamma_{A2}=\Gamma_{A3}=0$), we simply get $\langle x_{A}|\Psi_{A}\rangle =e^{jk_{0}x}$. In this case, other outcomes have zero probabilities. Equations (10) and (11) don’t contain the $e^{jk_{0}x}$ term because initially there is no any incident photon in the waveguides *B* and *C*. The moduli of the coordinates difference simply arise from the integrals, as for example it was shown in [14]. Thus, these moduli have a clear meaning because their sign defines transmission and reflection coefficients. For example, if we are interested in the reflection coefficient from Equation (9) (or in other words, in probability to find photon in the left side of qubit Q3 in *A* waveguide), we just set the following conditions:

(18) $$x<x_{1}^{A}\Rightarrow \left\{\begin{array}{c} e^{jk_{0}\left|x-x_{2}^{A}\right|}=e^{-jk_{0}x}\cdot e^{jk_{0}x_{2}^{A}};\\ e^{jk_{0}\left|x-x_{1}^{A}\right|}=e^{-jk_{0}x}\cdot e^{jk_{0}x_{1}^{A}}; \end{array}\right.$$

where $e^{-jk_{0}x}$ term describes the wave propagating in a left direction. According to these disclosures (18), we write the wave function as follows:

(19) $$\begin{array}{c} \langle x_{A}|\Psi_{A}\rangle =e^{jk_{0}x}-j\sqrt{\Gamma_{A2}}\cdot e^{-jk_{0}x}\left(R_{22}\sqrt{\Gamma_{A2}}e^{2jk_{0}x_{2}^{A}}+R_{32}\sqrt{\Gamma_{A3}}e^{jk_{0}\left(x_{2}^{A}+x_{1}^{A}\right)}\right)\\ -j\sqrt{\Gamma_{A3}}\cdot e^{-jk_{0}x}\left(R_{23}\sqrt{\Gamma_{A2}}e^{jk_{0}\left(x_{2}^{A}+x_{1}^{A}\right)}+R_{33}\sqrt{\Gamma_{A3}}e^{2jk_{0}x_{1}^{A}}\right). \end{array}$$

Then, by a natural defining reflection coefficient as a ratio of counter propagating wave to direct propagating wave, we get a reflection coefficient as:

(20) $$r_{AA}=-j\sqrt{\Gamma_{A2}}\left(R_{22}\sqrt{\Gamma_{A2}}e^{2jk_{0}x_{2}^{A}}+R_{32}\sqrt{\Gamma_{A3}}e^{jk_{0}\left(x_{2}^{A}+x_{1}^{A}\right)}\right)-j\sqrt{\Gamma_{A3}}\left(R_{23}\sqrt{\Gamma_{A2}}e^{jk_{0}\left(x_{2}^{A}+x_{1}^{A}\right)}+R_{33}\sqrt{\Gamma_{A3}}e^{2jk_{0}x_{1}^{A}}\right),$$

or, controversially, we can set $x>x_{2}^{A}$, and get the transmission coefficient in the waveguide *A*:

(21) $$\begin{array}{c} t_{AA}=1-j\sqrt{\Gamma_{A2}}\left(R_{22}\sqrt{\Gamma_{A2}}+R_{32}\sqrt{\Gamma_{A3}}e^{jk_{0}\left(x_{2}^{A}-x_{1}^{A}\right)}\right)\\ -j\sqrt{\Gamma_{A3}}\left(R_{23}\sqrt{\Gamma_{A2}}e^{jk_{0}\left(x_{1}^{A}-x_{2}^{A}\right)}+R_{33}\sqrt{\Gamma_{A3}}\right). \end{array}$$

These Equations (20) and (21) can be transformed to well-known results in the limit cases: (1) only coupling Q3 to *A* waveguide is non-zero and (2) only couplings of Q3 and Q2 to *A* waveguide are non-zero. In the first limit, we get the transmission and reflection coefficients demonstrated experimentally by Astafiev et al. [35]:

$$\begin{array}{cc} r_{AA} & =\frac{-j\Gamma_{A3}e^{2jk_{0}x_{1}^{A}}}{\omega -\Omega_{3}+j\Gamma_{A3}};\\ t_{AA} & =1-\frac{j\Gamma_{A3}}{\omega -\Omega_{3}+j\Gamma_{A3}} \end{array}$$

because, in this case, the effective Hamiltonian matrix (8) becomes diagonal. Here, it is obvious that at the frequency equal to $\Omega_{3}$ the reflection coefficient is equal to unity, and this fact is caused by the interference of the reflected and initial wave functions. It demonstrates the functionality of qubits Q3 and Q4; they serve as the additional scatterers to create appropriate interference conditions for the routing. The second limit leads to the results presented in [14,36,37]. The conditions below define the reflection and transmission when the photon after the scattering could be found at *B* or *C* waveguide, respectively:

(22) $$\begin{array}{c} x<x_{1}^{B}\;\;\;\;\mathrm{and}\;\;\;\;x>x_{2}^{B},\\ x<x_{1}^{C}\;\;\;\;\mathrm{and}\;\;\;\;x>x_{2}^{C}. \end{array}$$

If we don’t omit the indices of *x*, for example, when we apply the conditions (22) to Equations (10) and (11), we get a relation like $\frac{e^{jk_{0}x_{\left(B,C\right)}}}{e^{jk_{0}x_{A}}}\equiv e^{j\phi }$ because, for the photon initially sent to the *A* waveguide, we should normalize functions (10) and (11) to initial wave $e^{jk_{0}x_{A})}$. This element simply adds some phase shift ϕ for a scattered photon, and this fact is natural due to some phase incursion while the propagating in “perpendicular” to the waveguide direction. At Appendix A, we introduced the conditions for one-dimensionality, based on neglecting these phase incursions. However, in general, all Equations (9)–(17) consider these phase incursions.

Henceforward, we will specify $r_{fin-in}$ as the reflection coefficient, describing the probability to find the photon on the final (index fin) waveguide’s left side, when initially the photon was sent in the initial waveguide (index in). These initial and final waveguides refer to A,B,C waveguides, which could contain photons before and after scattering as well. The transmission coefficient $t_{fin-in}$ is specified with the same sense. In some simplification, the difference between the reflection and transmission is just a question about left or right sides in Figure 1. One can see that these coefficients (20) and (21) depend on the distance between qubits Q3 and Q2, and it results from the retardation effect (it was described in [14]). However, the more intriguing fact is that they both depend now on the distances between other qubits because each term contains this information from the effective Hamiltonian’s inversion (8). It is a direct manifestation of absolute quantum interferences between different wave functions, which are considered in all orders of interaction in a frame of the method. It can be shown by a direct substitution that, for each initial state manifold, the normalization condition is always satisfied:

(23) $$\sum_{J=A,B,C}{\left|t_{J-in}\right|}^{2}+{\left|r_{J-in}\right|}^{2}=1.$$

The calculations of all transmission and reflection amplitudes are presented in Appendix B. In the following section, we analyze these solutions and the router’s performance.

## 4. Simulations and Functionality of the Router

Because the router has a symmetry relative to *B* waveguide, it is intuitive to set the following equalities between distances $l_{23}=l_{14}=l_{side}$ (see Appendix A) and couplings $\Gamma_{A3}=\Gamma_{A2}=\Gamma_{C1}=\Gamma_{C4}=\Gamma_{side}$ and $\Gamma_{B1}=\Gamma_{B2}=\Gamma_{central}$. In addition, it is possible to introduce relations between the central waveguide and side waveguides parameters:

(24) $$\frac{\Gamma_{side}}{\Gamma_{central}}=\beta .$$

To simplify the optimization processes, we define non-dimensional distances:

(25) $$\begin{array}{c} \frac{\Theta_{N}}{\nu_{g}}l_{side}=L_{side},\\ \frac{\Theta_{N}}{\nu_{g}}l_{12}=L_{12}, \end{array}$$

where we introduced some fixed normalization frequency $\Theta_{N}$. This introduction leads to a substituting:

$$k_{0}=\frac{\omega }{\nu_{g}}\frac{\Theta_{N}}{\Theta_{N}}=\frac{\omega }{\Theta_{N}}k_{\Theta }.$$

The optimal distances have been found semi numerically by Quasi-Newton methods for extremum search with initial conditions defined by the following assumptions:

- central waveguide should introduce a minimum of phase increasing because the signal between $Q_{1}$ and $Q_{2}$ has minimal opportunity to escape in comparison to qubits $Q_{3}$ and $Q_{4}$, and we should not provide strong interference conditions in this region (for flexibility)
- the couplings of qubits $Q_{1}$ and $Q_{2}$ to the central waveguide should be greater than other couplings because the waveguide *B* provides intermediate interaction between *A* and *C* waveguides.

The proposed scheme can be used in two different configurations: as four-port router (when the central waveguide becomes auxiliary); and as the six-port device. For the first case, the system parameters are described in Table 1, and the router provides unity routing quality between waveguides *A* and *C*. We have only found such sets of preset parameters (distances between qubits, coupling strengths, etc.) that can provide the maximum not for all probabilities (for example, just for two ports of *B* waveguide), making tuning impossible for each probability by controllable parameters (qubits’ excitation frequency) in situ.

**Figure 2:**
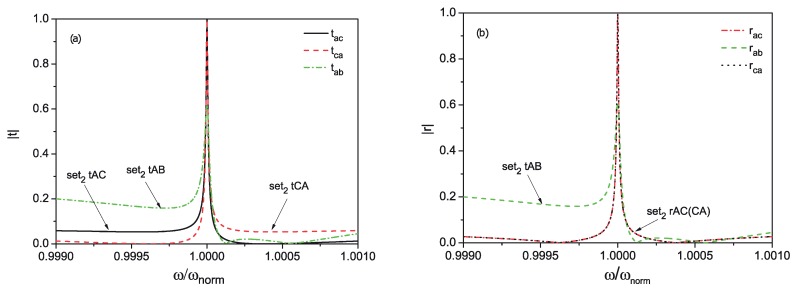
Transmission (**a**) and reflection (**b**) coefficients for the four-port router with the following parameters: β=3.4, $L_{side}=0.028$, $L_{12}=2\pi$, $\Theta_{N}$ is set to 5 GHz, $\Gamma_{central}=10$ MHz. The sets are described in Table 1.

From Figure 2, it is obvious that the probability to route the photon into the *B* waveguide cannot be more than 0.6 in the four-port configuration. Moreover, such configuration strongly depends on photon frequency, and it was found that it has a bandwidth of about 60 MHz (losing 15% of probability), repeated around the frequencies $\Theta_{x}=2n\Theta_{N},n=1,2,\cdots$. However, at least it can be used as a four-port device with tunable bandwidth with the unity routing efficiency.

**Figure 3:**
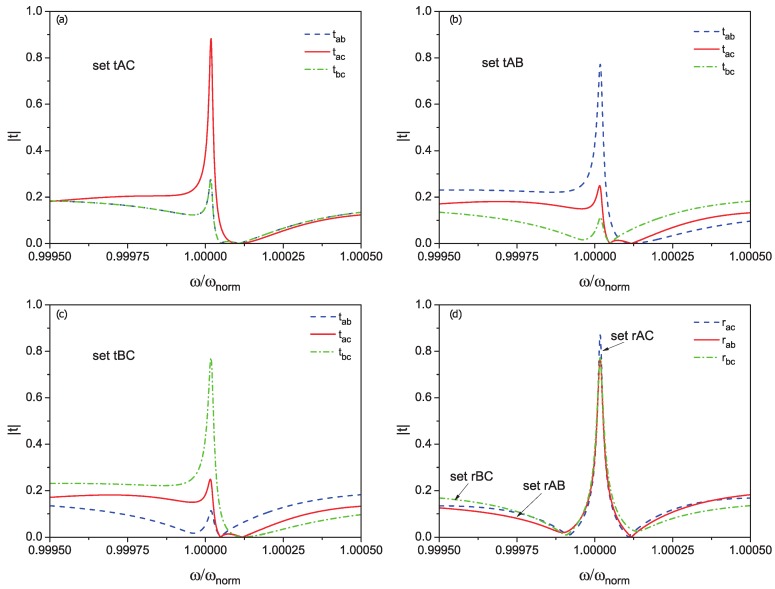
The six-port device’s tunability of routing by setting qubits’ excitations frequencies (set fIJ refers to combinations of to provide maximum transmission (f = t) or reflection coefficients (f = r) between waveguides *I* and *J* (I,J=A,B,C). Tranmission (**a**–**c**) and reflection (**d**) coefficients dependencies are shown. Parameters are the following: β=0.2, $L_{side}=\pi /30$, $L_{12}=0.01\pi$, $\Theta_{N}$ is set to 5GHz, $\Gamma_{central}=10$ MHz. The sets are described in Table 2.

We have found such parameters, which allow for using all six of the ports. These parameters are listed in Table 2. All transmission and reflection coefficients are defined exactly in Appendix B. In Figure 3, transmission (a, b, c) and reflection (d) coefficients for different combinations of qubits frequencies are shown.

One sees that the router could provide tunable probabilities in a range of 0.8 just by tuning the qubits’ frequencies. In addition, it is worth mentioning that, if we set all distances to be equal to zero, it will be impossible to tune the probabilities more than 0.5. It is an indirect manifestation that the routing is based on the interference and retardation effect, which are naturally described by the non-Hermitian Hamiltonian approach.

From Table 2, it is seen that the minimal difference between qubits’ excitation frequencies is around 200 kHz, and this can be easily provided by modern superconducting control schemes. We have checked that such sets could be found in a range of photon frequency from 2 GHz to 15 GHz (with fixed normalization $\Theta_{N}=5\;$ GHz, or put it another way with fixed distances) without any significant loss of the maximum probability amplitude.

The easiest control could be provided for scatterings without waveguide changing (photon stays at the same waveguide, in which it has been sent), and it is naturally obvious. For example, when qubits are not in the resonance with the photon, the last simply goes through the system without scatterings. One important thing should be mentioned about the relaxation rates of qubits. Of course, the existence of other quantum channels should decrease the probabilities to find a photon at the waveguides, but it is enough to provide coupling relation $\frac{\Gamma_{side}}{\gamma }\geq 10$ where γ is the maximal relaxation rate. This relation has been checked by numerical simulations with a substitution $\Omega_{i}\to \Omega_{i}-j\gamma$ because it is legal for single photon scattering and in the absence of a common thermal bath. A more accurate approach should include thermal baths to consider the relaxations.

A follow-up study should include expanded space of states, which are considered in the frame of the method. It is necessary in a problem of many photon scattering—for example, when two-excitation states are included. In this case, an opportunity to create and tune not only superposition states after scattering like $\alpha |k_{M}\rangle ⊗|G\rangle +\beta |k_{N}\rangle ⊗|G\rangle$ appears; here, M,N=A,B,C. Moreover, some Bell states (for example, like a $\alpha |k_{M}\rangle ⊗|g_{1},g_{2},e_{3},g_{4}\rangle +\beta |k_{N}\rangle ⊗|g_{1},g_{2},g_{3},e_{4}\rangle$ ) could be realized, if only one photon will escape the system after two-photon scattering.

**Figure 4:**
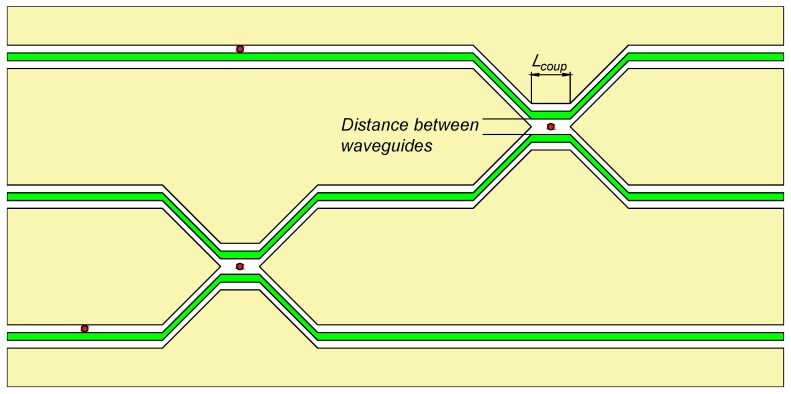
Sketch of the router topology based on a coplanar power divider scheme. Waveguides depicted as green lines, qubits’ positions are red, coplanar ground plates are yellow.

The proposed device might be realized in solid-state superconducting systems. For example, one can use coplanar waveguides in a power–divider configuration, where the coupler length is short enough to prevent direct coupling between them in a frequency range of interest (see Figure 4). The coupler length should be $L_{coup}=100\;\mu m$, which is from one point of view enough to place a superconducting qubit and, from another point of view, the direct coupling between the waveguides will be significant for the frequencies near $f_{coup}\approx \frac{c}{L_{coup}\sqrt{8\left(1+\varepsilon_{r}\right)}}\approx 300\;\mathrm{GHz}$, where we considered a silicon substrate with $\varepsilon_{r}=11.2$ [38]. The best candidates, as we suppose, are flux, gatemon, or X-mon qubits. For the flux qubit, several on-chip gap-tuning schemes were proposed [39,40,41] and, for gatemon qubits, the ability of gap tuning by local voltage on-chip gate was demonstrated recently [42]. The typical sizes of X-mon qubits allow for placing them between the coplanar waveguides.

It might be that the found parameters are not the global optimum. Nevertheless, the obtained equations could be used in optimization algorithms, and this is an important advantage of our method over numerical solutions.

## 5. Conclusions

In this work, we have shown the implementation of the non-Hermitian Hamiltonian approach to design single-photon quantum circuits. As an example, we offered the design of the single photon router and presented the calculation of its performance. The obtained equations are quite general, taking into account the spread of artificial atoms parameters as well as all distances between them. We have shown that the wavefunctions have the clear interpretation in a sense of the system’s functionality. Corresponding limiting cases demonstrate the validity of our approach. Analytical expressions for probabilities to detect a single photon at each waveguide have been derived. In the frame of the non-Hermitian approach, we showed that the routing arises naturally due to wave functions’ interference. We additionally note that the proposed and calculated device has the largest number of ports in comparison with known ones. We showed that our method accounts for the interaction in all orders of coupling strength between qubits and a scattering photon. This scheme has two different operating modes: (i) a six-port non-symmetric router and (ii) a four-port symmetric device.

For the narrowband regime, we reduce the number of operating ports to four ports. We have shown that the probability to detect a photon in each port can be set to near unity by an appropriate tuning of the qubits’ excitation frequency. For the wideband regime, we numerically found a set of optimal parameters, which allow for tuning a routing quality of more than 0.75 for each of the six ports (up to this time, it is the highest number of ports for proposed single photon routers). The proposed device fulfills the general requirements for the quantum single photon router and could be realized in a frame of existing solid-state technology. Moreover, considering potential application of the non-Hermitian Hamiltonian approach for cQED, a further study is required to generalize this method to multilevel atoms and scattering problems of many photons.

## Figures and Tables

**Table 1:** Example sets for tuning the routing of the four-port device.

	$\Omega_{1}-\Theta_{N}$, MHz	$\Omega_{2}-\Theta_{N}$, MHz	$\Omega_{3}-\Theta_{N}$, MHz	$\Omega_{4}-\Theta_{N}$, MHz
$set_{2}\;tAC$	−0.017	−0.024	1.901	1.898
$set_{2}\;tCA$	1.884	1.879	−0.0033	−0.0077
$set_{2}\;tAB$	2.193	−2.155	2.811	0.939
$set_{2}\;rAC$	1.883	−0.0076	1.886	−0.0067

**Table 2:** Example sets for tuning the routing of the six-port device.

	$\Omega_{1}-\Theta_{N}$, MHz	$\Omega_{2}-\Theta_{N}$, MHz	$\Omega_{3}-\Theta_{N}$, MHz	$\Omega_{4}-\Theta_{N}$, MHz
settAC	0.339	0.337	0.53	0.53
settAB	1.463	−0.542	0.581	0.239
settBC	−0.545	1.465	0.239	0.581
setrAC	0.893	0.334	0.534	−0.048
setrAB	−0.234	1.13	0.581	0.239
setrAC	0.086	1.479	0.239	−0.096

**Figure A1:**
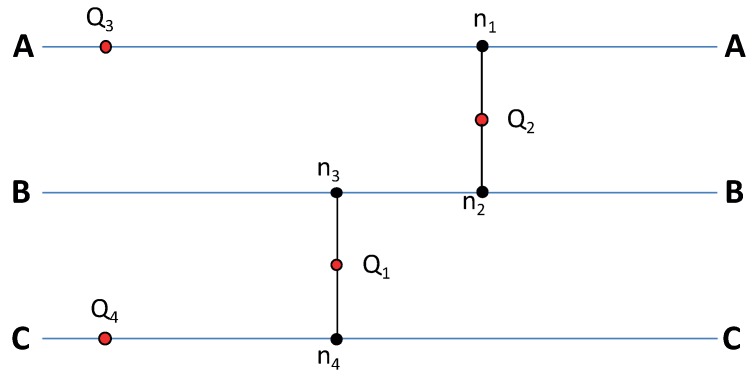
The scheme of quantum router: simplified diagram of the system.

**Figure A2:**
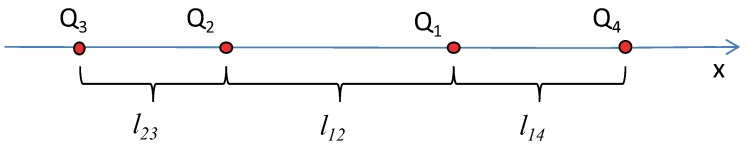
One-dimensional presentation of the system.

## Appendix A. Conditions of One-Dimensionality

Here, we start our reasoning from the definition of distances between the nodes $n_{i}$ in Figure A1, as it is shown in Table A1.

Just for the sake of simplicity, let sets $d_{Q_{2}-n_{1}},d_{Q_{2}-n_{2}},d_{Q_{1}-n_{3}},d_{Q_{1}-n_{4}}$ be equal to each other and assign this distance as δ. In this case, we can define distances $l_{mn}$ between $m^{th}$ and $n^{th}$ qubits as:

$$\begin{array}{c} \begin{array}{c} l_{12}=d_{n_{2}-n_{3}}+2\delta ;\\ l_{13}=d_{Q_{3}-n_{1}}+d_{n_{2}-n_{3}}+2\delta ;\\ l_{14}=d_{Q_{4}-n_{4}}+\delta ;\\ l_{23}=d_{Q_{3}-n_{1}}+\delta ;\\ l_{24}=d_{n_{2}-n_{3}}+d_{Q_{4}-n_{4}}+3\delta ;\\ l_{34}=d_{Q_{3}-n_{1}}+d_{n_{2}-n_{3}}+d_{Q_{4}-n_{4}}+4\delta . \end{array} \end{array}$$

One-dimensionality is introduced as the following conditions:

(A1) $$4\delta <<d_{Q_{3}-n_{1}},d_{n_{2}-n_{3}},d_{Q_{4}-n_{4}}.$$

These conditions could be easily realized in solid-state superconducting chips because distances like δ usually should be several hundreds of micrometers, while other significant lengths are typically in a centimeter range. The inequality (A1) leads us to the following relations:

$$\begin{array}{c} \begin{array}{c} l_{12}\approx d_{n_{2}-n_{3}};\\ l_{13}\approx d_{Q_{3}-n_{1}}+d_{n_{2}-n_{3}};\\ l_{14}\approx d_{Q_{4}-n_{4}};\\ l_{23}\approx d_{Q_{3}-n_{1}};\\ l_{24}\approx d_{n_{2}-n_{3}}+d_{Q_{4}-n_{4}};\\ l_{34}\approx d_{Q_{3}-n_{1}}+d_{n_{2}-n_{3}}+d_{Q_{4}-n_{4}}, \end{array} \end{array}$$

where all significant lengths are listed. It directly leads to the scheme presented in Figure 1 being able to be transformed into a pseudo-one-dimensional axis, as shown in Figure A2.

We can specify the zero coordinate point of this pseudo-axis as the middle point between $Q_{2}$ and $Q_{1}$. In this case, we redefine the qubits’ coordinates as follows:

(A2) $$\begin{array}{c} x_{1}^{A}=-l_{23}-\frac{1}{2}l_{12};\\ x_{2}^{A}=x_{2}^{B}=-\frac{1}{2}l_{12};\\ x_{1}^{B}=x_{2}^{C}=\frac{1}{2}l_{12};\\ x_{1}^{C}=l_{14}+\frac{1}{2}l_{12}. \end{array}$$

These equalities are used to disclose the modulus signs in Equations (9)–(17). However, we should mention that the final results don’t depend on the zero coordinate choices.

**Table A1:** Definitions of distances between qubits.

$d_{Q_{3}-n_{1}}$	distance between $Q_{3}$ and node $n_{1}$
$d_{Q_{2}-n_{1}}$	distance between $Q_{2}$ and node $n_{1}$
$d_{Q_{2}-n_{2}}$	distance between $Q_{2}$ and node $n_{2}$
$d_{n_{2}-n_{3}}$	distance between nodes $n_{2}$ and $n_{3}$
$d_{Q_{1}-n_{3}}$	distance between $Q_{1}$ and node $n_{3}$
$d_{Q_{1}-n_{4}}$	distance between $Q_{1}$ and node $n_{4}$
$d_{Q_{4}-n_{4}}$	distance between $Q_{4}$ and node $n_{4}$

## Appendix B. Scattering Parameters

To define scattering parameters, we should correctly disclose moduli signs in Equations (9)–(17). We do it by definitions (18) and (22) and get a set of 18 scattering amplitudes. For initial photon propagating in the *A* waveguide, we have three transmission amplitudes:

(A3) $$t_{AA}=1-j\sqrt{\Gamma_{A2}}\left(R_{22}\sqrt{\Gamma_{A2}}+R_{32}\sqrt{\Gamma_{A3}}e^{jk_{0}l_{23}}\right)-j\sqrt{\Gamma_{A3}}\left(R_{23}\sqrt{\Gamma_{A2}}e^{-jk_{0}l_{23}}+R_{33}\sqrt{\Gamma_{A3}}\right),$$

(A4) $$t_{AB}=-j\sqrt{\Gamma_{A2}}\left(R_{12}\sqrt{\Gamma_{B1}}e^{-jk_{0}l_{12}}+R_{22}\sqrt{\Gamma_{B2}}\right)-j\sqrt{\Gamma_{A3}}\left(R_{13}\sqrt{\Gamma_{B1}}e^{-jk_{0}\left(l_{12}+l_{23}\right)}+R_{23}\sqrt{\Gamma_{B2}}e^{-jk_{0}l_{23}}\right),$$

(A5) $$t_{AC}=-j\sqrt{\Gamma_{A2}}\left(\begin{array}{c} R_{12}\sqrt{\Gamma_{C1}}e^{-jk_{0}l_{12}}\\ +R_{42}\sqrt{\Gamma_{C4}}e^{-jk_{0}\left(l_{12}+l_{14}\right)} \end{array}\right)-j\sqrt{\Gamma_{A3}}\left(\begin{array}{c} R_{13}\sqrt{\Gamma_{C1}}e^{-jk_{0}\left(l_{23}+l_{12}\right)}\\ +R_{43}\sqrt{\Gamma_{C4}}e^{-jk_{0}\left(l_{12}+l_{23}+l_{14}\right)} \end{array}\right),$$

and three reflection amplitudes:

(A6) $$r_{AA}=-j\sqrt{\Gamma_{A2}}\left(\begin{array}{c} R_{22}\sqrt{\Gamma_{A2}}e^{-jk_{0}l_{12}}\\ +R_{32}\sqrt{\Gamma_{A3}}e^{-jk_{0}\left(l_{12}+l_{23}\right)} \end{array}\right)-j\sqrt{\Gamma_{A3}}\left(\begin{array}{c} R_{23}\sqrt{\Gamma_{A2}}e^{-jk_{0}\left(l_{12}+l_{23}\right)}\\ +R_{33}\sqrt{\Gamma_{A3}}e^{-jk_{0}\left(l_{12}+2l_{23}\right)} \end{array}\right),$$

(A7) $$r_{AB}=-j\sqrt{\Gamma_{A2}}\left(R_{12}\sqrt{\Gamma_{B1}}+R_{22}\sqrt{\Gamma_{B2}}e^{-jk_{0}l_{12}}\right)-j\sqrt{\Gamma_{A3}}\left(R_{13}\sqrt{\Gamma_{B1}}e^{-jk_{0}l_{23}}+R_{23}\sqrt{\Gamma_{B2}}e^{-jk_{0}\left(l_{12}+l_{23}\right)}\right),$$

(A8) $$r_{AC}=-j\sqrt{\Gamma_{A2}}\left(R_{12}\sqrt{\Gamma_{C1}}+R_{42}\sqrt{\Gamma_{C4}}e^{jk_{0}l_{14}}\right)-j\sqrt{\Gamma_{A3}}\left(R_{13}\sqrt{\Gamma_{C1}}e^{-jk_{0}l_{23}}+R_{43}\sqrt{\Gamma_{C4}}e^{-jk_{0}\left(l_{23}-l_{14}\right)}\right).$$

Similarly, for initially photon propagating in the *B* waveguide, there are three transmission amplitudes:

(A9) $$t_{BA}=-j\sqrt{\Gamma_{B1}}\left(R_{21}\sqrt{\Gamma_{A2}}e^{jk_{0}l_{12}}+R_{31}\sqrt{\Gamma_{A3}}e^{jk_{0}\left(l_{12}+l_{23}\right)}\right)-j\sqrt{\Gamma_{B2}}\left(R_{22}\sqrt{\Gamma_{A2}}+R_{32}\sqrt{\Gamma_{A3}}e^{jk_{0}l_{23}}\right),$$

(A10) $$t_{BB}=1-j\sqrt{\Gamma_{B1}}\left(R_{11}\sqrt{\Gamma_{B1}}+R_{21}\sqrt{\Gamma_{B2}}e^{jk_{0}l_{12}}\right)-j\sqrt{\Gamma_{B2}}\left(R_{12}\sqrt{\Gamma_{B1}}e^{-jk_{0}l_{12}}+R_{22}\sqrt{\Gamma_{B2}}\right),$$

(A11) $$t_{BC}=-j\sqrt{\Gamma_{B1}}\left(R_{11}\sqrt{\Gamma_{C1}}+R_{41}\sqrt{\Gamma_{C4}}e^{-jk_{0}l_{14}}\right)-j\sqrt{\Gamma_{B2}}\left(R_{12}\sqrt{\Gamma_{C1}}e^{-jk_{0}l_{12}}+R_{42}\sqrt{\Gamma_{C4}}e^{-jk_{0}\left(l_{12}+l_{14}\right)}\right),$$

and three reflection amplitudes:

(A12) $$r_{BA}=-j\sqrt{\Gamma_{B1}}\left(R_{21}\sqrt{\Gamma_{A2}}+R_{31}\sqrt{\Gamma_{A3}}e^{-jk_{0}l_{23}}\right)-j\sqrt{\Gamma_{B2}}\left(R_{22}{\sqrt{\Gamma_{A2}}}^{-jk_{0}l_{12}}+R_{32}\sqrt{\Gamma_{A3}}e^{-jk_{0}\left(l_{23}+l_{12}\right)}\right),$$

(A13) $$r_{BB}=-j\sqrt{\Gamma_{B1}}\left(R_{11}\sqrt{\Gamma_{B1}}e^{jk_{0}l_{12}}+R_{21}\sqrt{\Gamma_{B2}}\right)-j\sqrt{\Gamma_{B2}}\left(R_{12}\sqrt{\Gamma_{B1}}+R_{22}\sqrt{\Gamma_{B2}}e^{-jk_{0}l_{12}}\right),$$

(A14) $$r_{BC}=-j\sqrt{\Gamma_{B1}}\left(R_{11}\sqrt{\Gamma_{C1}}e^{jk_{0}l_{12}}+R_{41}\sqrt{\Gamma_{C4}}e^{jk_{0}\left(l_{14}+l_{12}\right)}\right)-j\sqrt{\Gamma_{B2}}\left(R_{12}\sqrt{\Gamma_{C1}}+R_{42}\sqrt{\Gamma_{C4}}e^{jk_{0}l_{14}}\right).$$

For initial photon propagating in the *C* waveguide, transmission amplitudes are the following:

(A15) $$t_{CA}=-j\sqrt{\Gamma_{C1}}\left(\begin{array}{c} R_{21}\sqrt{\Gamma_{A2}}e^{jk_{0}l_{12}}\\ +R_{31}\sqrt{\Gamma_{A3}}e^{jk_{0}\left(l_{12}+l_{23}\right)} \end{array}\right)-j\sqrt{\Gamma_{C4}}\left(\begin{array}{c} R_{24}\sqrt{\Gamma_{A2}}e^{jk_{0}\left(l_{14}+l_{12}\right)}\\ +R_{34}\sqrt{\Gamma_{A3}}e^{jk_{0}\left(l_{14}+l_{12}+l_{23}\right)} \end{array}\right),$$

(A16) $$t_{CB}=-j\sqrt{\Gamma_{C1}}\left(R_{11}\sqrt{\Gamma_{B1}}+R_{21}\sqrt{\Gamma_{B2}}e^{jk_{0}l_{12}}\right)-j\sqrt{\Gamma_{C4}}\left(R_{14}\sqrt{\Gamma_{B1}}e^{jk_{0}l_{14}}+R_{24}\sqrt{\Gamma_{B2}}e^{jk_{0}\left(l_{14}+l_{12}\right)}\right),$$

(A17) $$t_{CC}=1-j\sqrt{\Gamma_{C1}}\left(R_{11}\sqrt{\Gamma_{C1}}+R_{41}\sqrt{\Gamma_{C4}}e^{-jk_{0}l_{14}}\right)-j\sqrt{\Gamma_{C4}}\left(R_{14}\sqrt{\Gamma_{C1}}e^{jk_{0}l_{14}}+R_{44}\sqrt{\Gamma_{C4}}\right),$$

and reflection amplitudes:

(A18) $$r_{CA}=-j\sqrt{\Gamma_{C1}}\left(R_{21}\sqrt{\Gamma_{A2}}+R_{31}\sqrt{\Gamma_{A3}}e^{-jk_{0}l_{23}}\right)-j\sqrt{\Gamma_{C4}}\left(R_{24}\sqrt{\Gamma_{A2}}e^{jk_{0}l_{14}}+R_{34}\sqrt{\Gamma_{A3}}e^{jk_{0}\left(l_{14}-l_{23}\right)}\right),$$

(A19) $$r_{CB}=-j\sqrt{\Gamma_{C1}}\left(R_{11}\sqrt{\Gamma_{B1}}e^{jk_{0}l_{12}}+R_{21}\sqrt{\Gamma_{B2}}\right)-j\sqrt{\Gamma_{C4}}\left(R_{14}\sqrt{\Gamma_{B1}}e^{jk_{0}\left(l_{14}+l_{12}\right)}+R_{24}\sqrt{\Gamma_{B2}}e^{jk_{0}l_{14}}\right),$$

(A20) $$r_{CC}=-j\sqrt{\Gamma_{C1}}\left(\begin{array}{c} R_{11}\sqrt{\Gamma_{C1}}e^{jk_{0}l_{12}}\\ +R_{41}\sqrt{\Gamma_{C4}}e^{jk_{0}\left(l_{14}+l_{12}\right)} \end{array}\right)-j\sqrt{\Gamma_{C4}}\left(\begin{array}{c} R_{14}\sqrt{\Gamma_{C1}}e^{jk_{0}\left(l_{14}+l_{12}\right)}\\ +R_{44}\sqrt{\Gamma_{C4}}e^{jk_{0}\left(2l_{14}+l_{12}\right)} \end{array}\right).$$

## Appendix C. Inverse Matrix R

Here, we present elements of inverse matrix *R*. Firstly, let’s rewrite the effective Hamiltonian (8) in a simpler form:

(A21) $${\hat{H}}_{eff}\left(\omega \right)=\left(\begin{array}{cccc} H_{11} & H_{12}\left(\omega \right) & 0 & H_{14}\left(\omega \right)\\ H_{12}\left(\omega \right) & H_{22} & H_{23}\left(\omega \right) & 0\\ 0 & H_{23}\left(\omega \right) & H_{33} & 0\\ H_{14}\left(\omega \right) & 0 & 0 & H_{44} \end{array}\right).$$

In this formulation of the effective Hamiltonian inverse matrix, *R* elements are:

(A22) $$R_{11}\left(\omega \right)=\frac{1}{D\left(\omega \right)}\left(\omega -H_{44}\right)\left(\left(\omega -H_{33}\right)\left(\omega -H_{22}\right)-H_{23}^{2}\left(\omega \right)\right),$$

(A23) $$R_{12}\left(\omega \right)=R_{21}\left(\omega \right)=\frac{1}{D\left(\omega \right)}H_{12}\left(\omega \right)\left(\omega -H_{33}\right)\left(\omega -H_{44}\right),$$

(A24) $$R_{13}\left(\omega \right)=R_{31}\left(\omega \right)=\frac{1}{D\left(\omega \right)}H_{12}\left(\omega \right)H_{23}\left(\omega \right)\left(\omega -H_{44}\right),$$

(A25) $$R_{14}\left(\omega \right)=R_{41}\left(\omega \right)=\frac{1}{D\left(\omega \right)}H_{14}\left(\omega \right)\left(\left(\omega -H_{33}\right)\left(\omega -H_{22}\right)-H_{23}^{2}\left(\omega \right)\right),$$

(A26) $$R_{22}\left(\omega \right)=\frac{1}{D\left(\omega \right)}\left(\omega -H_{33}\right)\left(\left(\omega -H_{11}\right)\left(\omega -H_{44}\right)-H_{14}^{2}\left(\omega \right)\right),$$

(A27) $$R_{23}\left(\omega \right)=R_{32}\left(\omega \right)=\frac{1}{D\left(\omega \right)}H_{23}\left(\omega \right)\left(\left(\omega -H_{11}\right)\left(\omega -H_{44}\right)-H_{14}^{2}\left(\omega \right)\right),$$

(A28) $$R_{24}\left(\omega \right)=R_{42}\left(\omega \right)=\frac{1}{D\left(\omega \right)}H_{12}\left(\omega \right)H_{14}\left(\omega \right)\left(\omega -H_{33}\right),$$

(A29) $$R_{33}\left(\omega \right)=\frac{1}{D\left(\omega \right)}\left[\begin{array}{c} \left(\omega -H_{11}\right)\left(\omega -H_{22}\right)\left(\omega -H_{44}\right)\\ -\left(\omega -H_{22}\right)H_{14}^{2}\left(\omega \right)-\left(\omega -H_{44}\right)H_{12}^{2}\left(\omega \right) \end{array}\right],$$

(A30) $$R_{34}\left(\omega \right)=R_{43}\left(\omega \right)=\frac{1}{D\left(\omega \right)}H_{12}\left(\omega \right)H_{14}\left(\omega \right)H_{23}\left(\omega \right),$$

(A31) $$R_{44}\left(\omega \right)=\frac{1}{D\left(\omega \right)}\left[\begin{array}{c} \left(\omega -H_{11}\right)\left(\omega -H_{22}\right)\left(\omega -H_{33}\right)\\ -\left(\omega -H_{11}\right)H_{23}^{2}\left(\omega \right)-\left(\omega -H_{33}\right)H_{12}^{2}\left(\omega \right) \end{array}\right],$$

where is determinant of R matrix, and can be written as follows:

$$\begin{array}{c} D\left(\omega \right)=H_{14}^{2}\left(\omega \right)H_{23}^{2}\left(\omega \right)\\ -\left(\omega -H_{44}\right)\left[\left(\omega -H_{11}\right)H_{23}^{2}\left(\omega \right)+\left(\omega -H_{33}\right)H_{12}^{2}\left(\omega \right)\right]\\ +\left(\omega -H_{22}\right)\left(\omega -H_{33}\right)\left[\left(\omega -H_{11}\right)\left(\omega -H_{44}\right)-H_{14}^{2}\left(\omega \right)\right]. \end{array}$$
